# Laparoscopic nerve‑sparing radical hysterectomy for the treatment of cervical cancer: a meta-analysis of randomized controlled trials

**DOI:** 10.1186/s12957-021-02408-x

**Published:** 2021-10-18

**Authors:** Linlin Ma, Qiwei Li, Ying Guo, Xiaoyu Tan, Mengying Wang, Qi Qi

**Affiliations:** 1 Department of Obstetrics and Gynecology, The First Affiliated Hospital of Heilongjiang University of Chinese Medicine, No. 26 Heping Road, Xiangfang District, Harbin City, 150040 Heilongjiang Province China; 2 Department of Obstetrics and Gynecology, Harbin Fifth Hospital, Harbin City, China

**Keywords:** Nerve sparing, Radical hysterectomy, Cervical cancer, Surgery, Treatment, Meta-analysis

## Abstract

**Background:**

The effects and safety of laparoscopic nerve‑sparing radical hysterectomy (LNSRH) and laparoscopic radical hysterectomy (LRH) in cervical cancer treatment remain unclear. This article aims to evaluate the role of LNSRH versus LRH in the treatment of cervical cancer. This is because the updated meta-analysis with synthesized data may provide more reliable evidence on the role of LNSRH and LRH.

**Methods:**

We searched Pubmed et al. databases for randomized controlled trials (RCTs) involving laparoscopic nerve‑sparing radical hysterectomy (LNSRH) and laparoscopic radical hysterectomy (LRH) for cervical cancer treatment from the inception of databases to June 15, 2021. The RevMan 5.3 software was used for data analyses. This meta-analysis protocol had been registered online (available at: https://inplasy.com/inplasy-2021-9-0047/).

**Results:**

Thirteen RCTs involving a total of 1002 cervical cancer patients were included. Synthesized results indicated that the duration of surgery of the LNSRH group was significantly longer than that of the LRH group [SMD 1.11, 95% CI (0.15 ~ 2.07), *P* = 0.02]. The time to intestinal function recovery [SMD −1.27, 95% CI (−1.84 ~ −0.69), *P* < 0.001] and the time to postoperative urinary catheter removal of the LNSRH group [SMD −1.24, 95% CI (−1.62 ~ −0.86), P < 0.001] were significantly less than that of the LRH group. There were no significant differences in the estimated blood loss [SMD 0.10, 95% CI (−0.14 ~ 0.34), *P* = 0.41], the length of parauterine tissue resection [SMD −0.10, 95% CI (−0.25 ~ 0.05), *P* = 0.19], length of vaginal excision [SMD 0.04, 95% CI (−0.26 ~ 0.34), *P* = 0.78], and incidence of intraoperative adverse events [RR 0.97, 95% CI (0.44 ~ 2.13), *P* = 0.94] between the LNSRH group and the LRH group.

**Conclusions:**

LNSRH significantly results in earlier bladder and bowel function after surgery. Limited by sample size, LNSRH should be considered with caution in the future.

## Background

Cervical cancer is the second leading cause of death among women followed by breast cancer [1]. There are about 500,000 new cases worldwide each year, of which 80% occur in developing countries [2]. Early cervical cancer can be treated with surgery or radiotherapy. At present, due to the unequal availability of radiotherapy and chemotherapy in China, early cervical cancer is still treated mainly by surgery [3]. Since Wertheim completed the first radical hysterectomy for cervical cancer in 1898, in the following 100 years, it has undergone improvements by Bonney, Meigs, Piver, for example, classic Piver type II or type III radical hysterectomy [4]. Surgery is currently the mainstay treatment in early stage of cervical cancer based on FIGO staging, but the concurrent complications of severe urinary, rectal, and sexual dysfunction occurring post surgery seriously affect the quality of life [5, 6]. Therefore, maximizing post-surgical quality of life without compromising the oncological outcome has become the focus of research.

Based on the understanding of tissue structure around the cervix and innervation, and the understanding of the shape, function, and innervation of the pelvic autonomic nerves, some scholars have proposed a radical resection of cervical cancer that preserves the pelvic nerves [7]. Laparoscopic radical hysterectomy (LRH) has the advantages of performing fine dissection, less intraoperative bleeding, superior surgical field visibility, less tissue trauma, and quicker recovery [8]. The widespread use in laparoscopic radical cervical cancer has enabled more doctors to master pelvic nerve-preserving radical cervical cancer. Conventional LRH leads to cutting of the pelvic splanchnic nerves as we dissect the uterosacral ligament, internal iliac lymphadenectomy, and the parametrial tissues where these nerves pass, resulting in bladder and bowel dysfunction. Laparoscopic nerve‑sparing radical hysterectomy (LNSRH) allows preservation of the pelvic visceral nerves during radical cervical cancer surgery [9, 10]. However, the effect and safety of radical cervical cancer surgery with pelvic nerve preservation are not yet fully understood [11]. Therefore, we aimed to conduct a meta-analysis of LNSRH to analyze the safety and effectiveness, and to provide reliable evidence for the treatment of cervical cancer.

## Methods

We performed and reported this meta-analysis according to the Preferred Reporting Items for Systematic Reviews and Meta-Analyses (PRISMA) [12]. This meta-analysis protocol had been registered online (available at: https://inplasy.com/inplasy-2021-9-0047/) with registered number: INPLASY202190047.

### Literature search

We searched Pubmed, Embase, Web of Science, Cochrane Library, Weipu database, Tsinghua Tongfang database, and China National Knowledge Infrastructure (CNKI) for randomized controlled trials (RCTs) involving independent LNSRH and LRH as the treatment of early stage of cervical cancer based on FIGO staging, the search time limit was from the inception of databases to June 15, 2021. The search strategies used in this present meta-analysis were as follows: (laparoscopic nerve-sparing radical hysterectomy) OR (LNSRH) OR (nerve sparing)) AND (laparoscopic radical hysterectomy) OR (LRH)) AND (cervical cancer). Besides, we checked and searched the reference lists of the RCTs and reviews that met the inclusion criteria of our study.

### Inclusion and exclusion criteria’s

The inclusion criteria for this meta-analysis were as follows: patients with cervical cancer; comparison of independently LNSRH and LRH in treatment of early stage of cervical cancer based on FIGO staging; RCT study design; related outcomes and complete data were reported. Studies were excluded if the study included only one surgical treatment group without a comparison design. We excluded the studies failing to report the basic study characteristics such as age, body mass index, FIGO stage. Reviews, editorials, guidelines, case reports, letters, and meeting papers were excluded.

### Literature screening and extraction

The full text of the selected literature was reviewed and data was tabulated. The extracted information included the setting, author, year of publication, research populations, the number of cases, treatment details including duration of surgery, estimated blood loss, length of parauterine tissue resection, length of vaginal excision, time to intestinal function recovery, time to postoperative urinary catheter removal, and the incidence of intraoperative adverse events.

### Evaluation of included RCTs

We used the Cochrane Collaborations risk of bias tool to assess the methodological quality and risk of bias of analyzed RCTs. Seven specific domains were evaluated in this tool: i.e., sequence generation, allocation concealment, blinding of participants and personnel, blinding of outcome assessment, incomplete outcome data, selective outcome reporting, and other issues. Each domain was rated as low, high, or unclear risk of bias according to the judgment criteria. The literature quality evaluation was independently completed by two literature review researchers. When the two of them disagreed, the third evaluator intervened and reached a consensus through discussion.

### Statistical analysis

All statistical analyses were performed using the RevMan 5.3 software. Data were used as input and double-checked by two authors. All the data syntheses and interpretations were also conducted by two authors to ensure the accuracy of the results. Binary outcomes were presented as Mantel–Haenszel-style risk ratio (RR) with 95% confidence intervals (CI). Continuous outcomes were presented as standardized mean differences (SMD). A fixed-effect model was applied in cases of homogeneity (*P* value of *χ*^2^ test > .10 and *I*^2^  <  50%), and a random-effect model was applied in cases of obvious heterogeneity (*P* value of *χ*^2^ test  > .10 and *I*^2^ ≥ 50%). Publication bias was assessed by funnel plots, and we conducted Egger regression test to evaluate the asymmetry. In this study, the difference was statistically significant with *P* < 0.05.

## Results

### Study selection

**Fig. 1:**
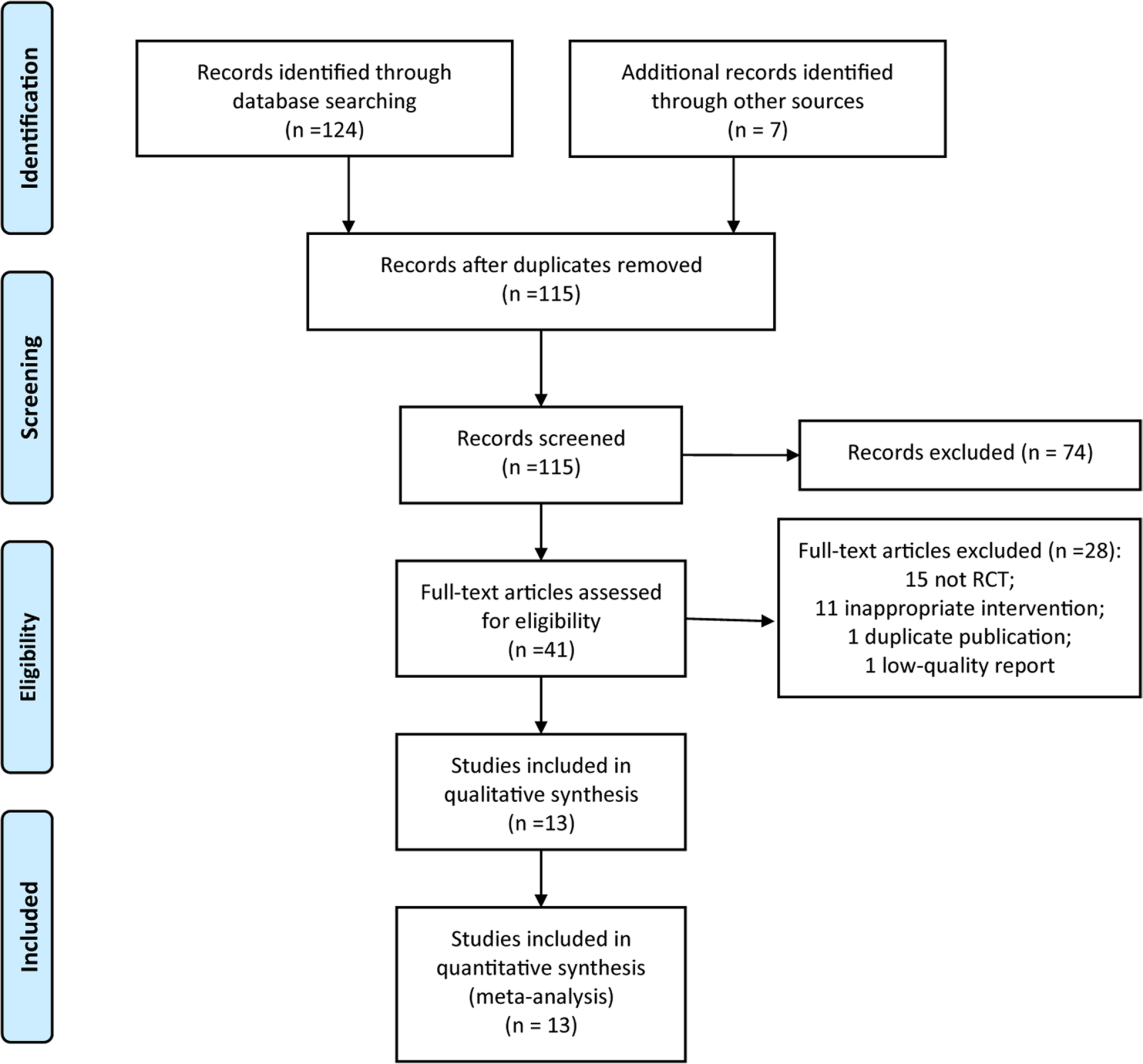
PRISMA flow diagram for study selection

The flow diagram of study selection process is shown in Fig. 1. A total of 131 reports were extracted from the initial searches. After the titles and abstracts were reviewed, 41 full-text reports were assessed for eligibility. Finally, 13 RCTs [13–25] were included for meta-analysis in this present meta-study.

### The characteristics and quality of included RCTs

**Fig. 2:**
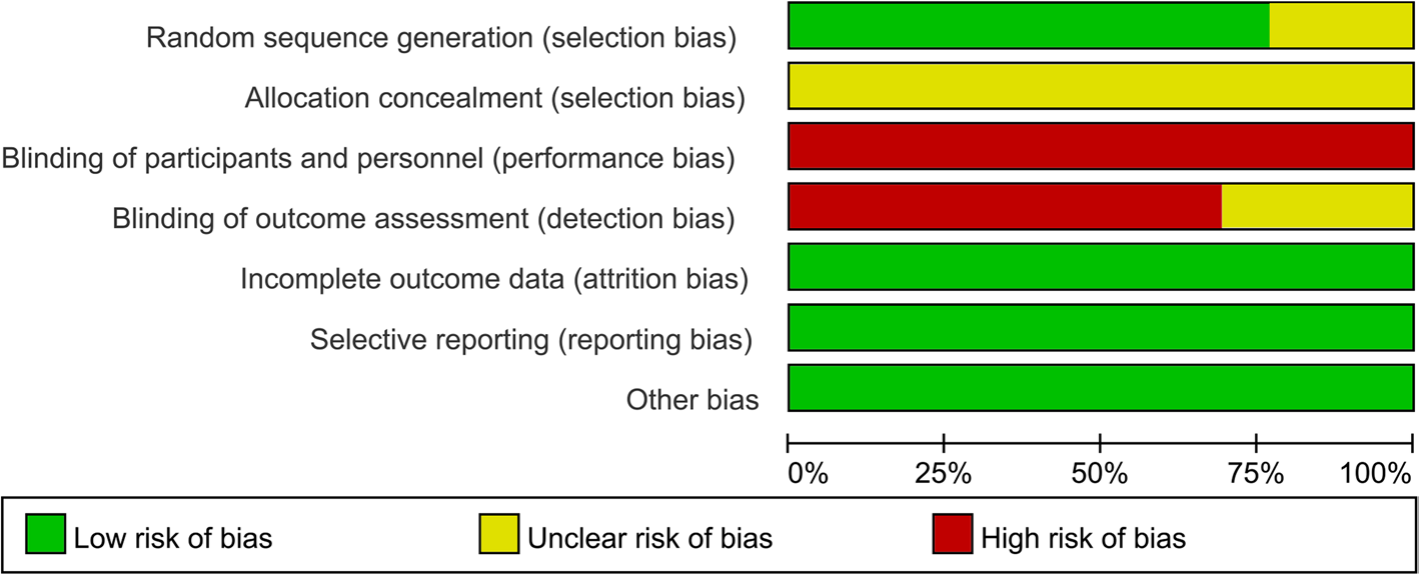
Risk of bias graph

**Fig. 3:**
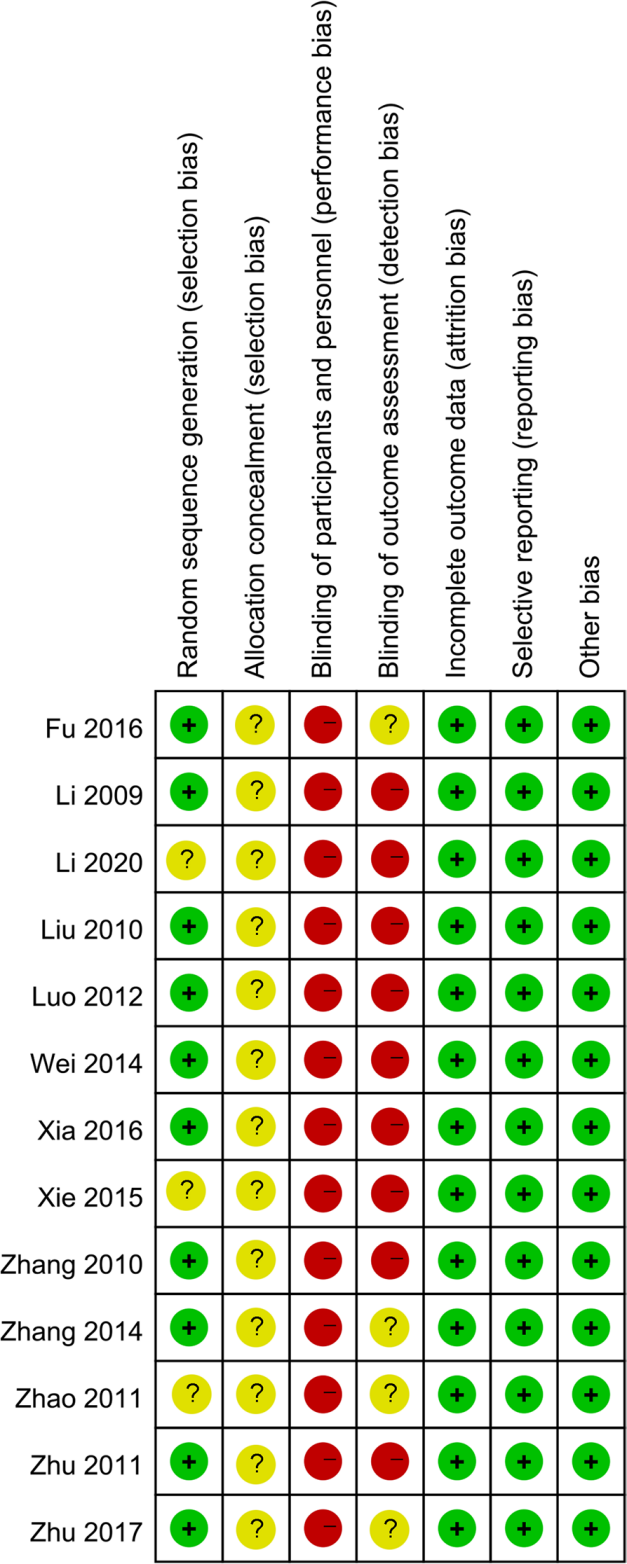
Risk of bias summary

The main characteristics of the included RCTs are shown in Table 1. All the 13 RCTs were conducted and reported in China. The 13 RCTs [13–25] included a total of 1002 cervical cancer patients, 489 of whom were treated with LNSRH, and 513 of whom were treated with LRH. The patients reported in the included studies had similar cancer FIGO stage, and the baseline data were comparable among the patients undergoing LNSRH versus LRH. The quality of included RCTs is indicated in Figs. 2 and 3.

**Table 1:** The characteristics of included RCTs

Study ID	Country	Sample size		Age (years)		BMI (kg/m^2^)		FIGO (IA2/IB1/IB2/IIA/IIB, *n*)		Neoadjuvant chemotherapy	
		LNSRH	LRH	LNSRH	LRH	LNSRH	LRH	LNSRH	LRH	LNSRH	LRH
Fu 2016	China	21	23	42.6 ± 8.9	43.1 ± 9.2	23.6± 3.8	23.6 ± 4.2	NA	NA	0	0
Li 2009	China	25	25	44.02 ± 9.15	43.87 ± 8.91	22.64 ± 3.78	23.11 ± 4.81	NA	NA	NA	NA
Li 2020	China	34	34	43. 19 ± 6. 98	43. 06 ± 6. 82	23. 57 ± 1. 78	23. 61 ± 1. 85	0/15/8/11/0	0/16/9/9/0	0	0
Liu 2010	China	41	46	42.5 ± 9.4	44.0 ± 8.0	23.3 ± 4.1	22.1 ± 5.2	0/7/16/18/0	0/9/15/22/0	NA	NA
Luo 2012	China	26	32	43.0 ± 10.0	44.0 ± 9.0	NA	NA	0/7/10/9/0	0/10/15/7/0	0	0
Wei 2014	China	32	20	41.93 ± 8.66	42.09±9.15	NA	NA	NA	NA	0	0
Xia 2016	China	116	119	42.32 ± 9.79	42.76±10.11	22.74 ± 3.12	22.18±4.01	NA	NA	NA	NA
Xie 2015	China	45	45	43.5 ± 3.8	43.4 ± 4.0	23.5 ± 2.1	23.6 ± 1.9	0/31/7/7/0	0/32/5/8/0	0	0
Zhang 2010	China	17	18	41.0 ± 8.0	39.0 ± 6.0	24.2 ± 3.5	24.4 ± 3.1	3/6/5/3/0	2/8/4/4/0	NA	NA
Zhang 2014	China	15	33	43.4 ± 11.2	48.3 ± 8.7	NA	NA	NA	NA	NA	8
Zhao 2011	China	17	13	39.7 ± 8.4	43.2 ± 9.4	NA	NA	7/9/0/1/0	4/4/0/5/0	0	0
Zhu 2011	China	38	43	43.0 ± 11.0	44.0 ± 10.0	NA	NA	15/20/0/3/0	17/22/0/4/0	0	0
Zhu 2017	China	62	62	42.0 ± 6.6	41.4 ± 6.4	NA	NA	0/33/11/18/0	0/38/7/17/0	NA	NA

### Synthesized outcomes

#### Estimated blood loss

**Fig. 4:**
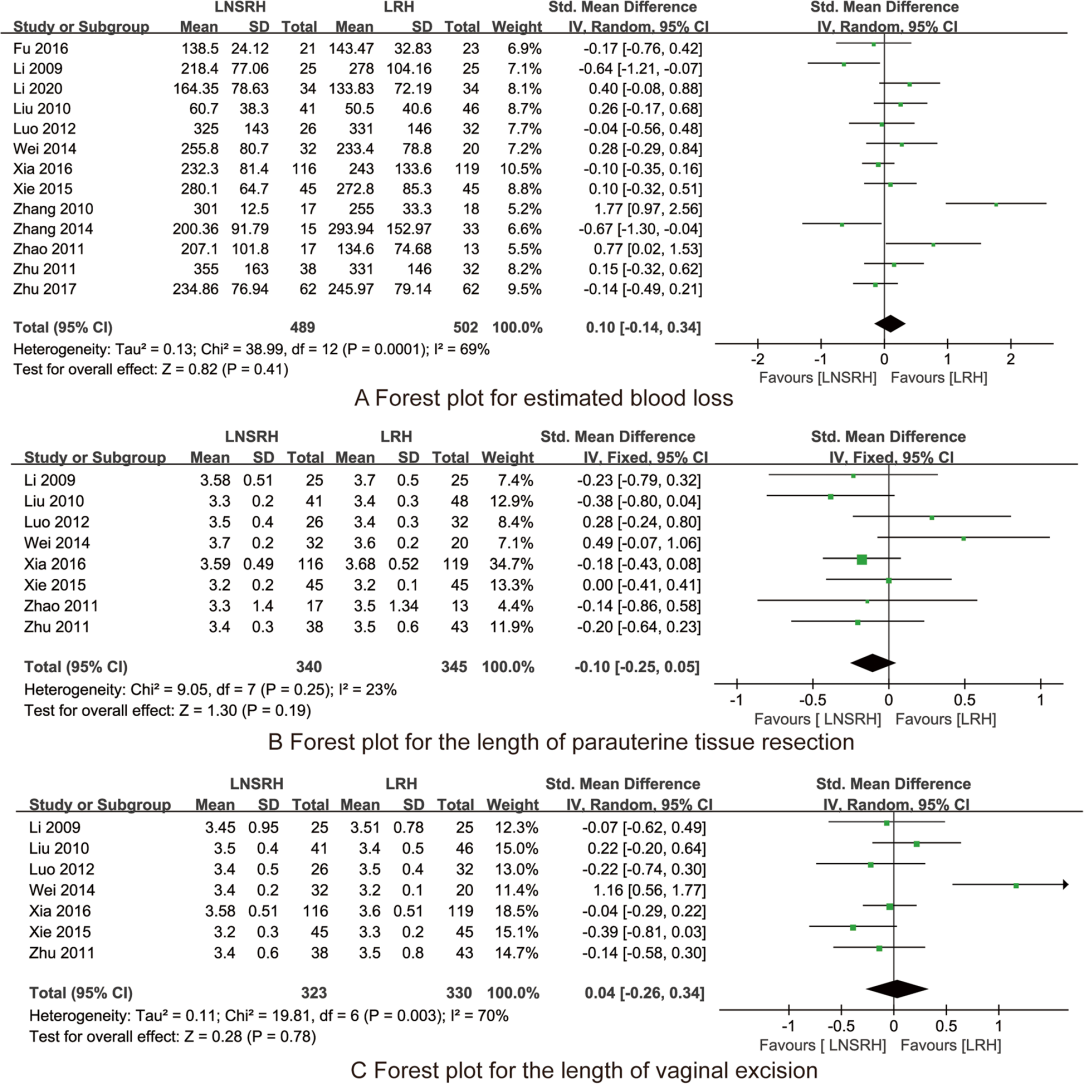
The forest plots for synthesized outcomes

Thirteen RCTs [13–25] reported the estimated blood loss between LNSRH group and the LRH group. As presented in Fig. 4A, there is no significant difference in the estimated blood loss between the LNSRH group and the LRH group [SMD 0.10, 95% CI (−0.14 ~ 0.34), *P* = 0.41].

#### Length of parauterine tissue resection

Eight RCTs [13, 14, 16, 18–20, 24, 25] reported the length of parauterine tissue resection between LNSRH group and the LRH group. As presented in Fig. 4B, there is no significant difference in the length of parauterine tissue resection between the LNSRH group and the LRH group [SMD −0.10, 95% CI (−0.25 ~ 0.05), *P* = 0.19].

#### The length of vaginal excision

Seven RCTs [13, 14, 17–20, 24] reported the length of vaginal excision between LNSRH group and the LRH group. As presented in Fig. 4C, there is no significant difference in the length of vaginal excision between the LNSRH group and the LRH group [SMD 0.04, 95% CI (−0.26 ~ 0.34), *P* = 0.78].

#### Duration of surgery

**Fig. 5:**
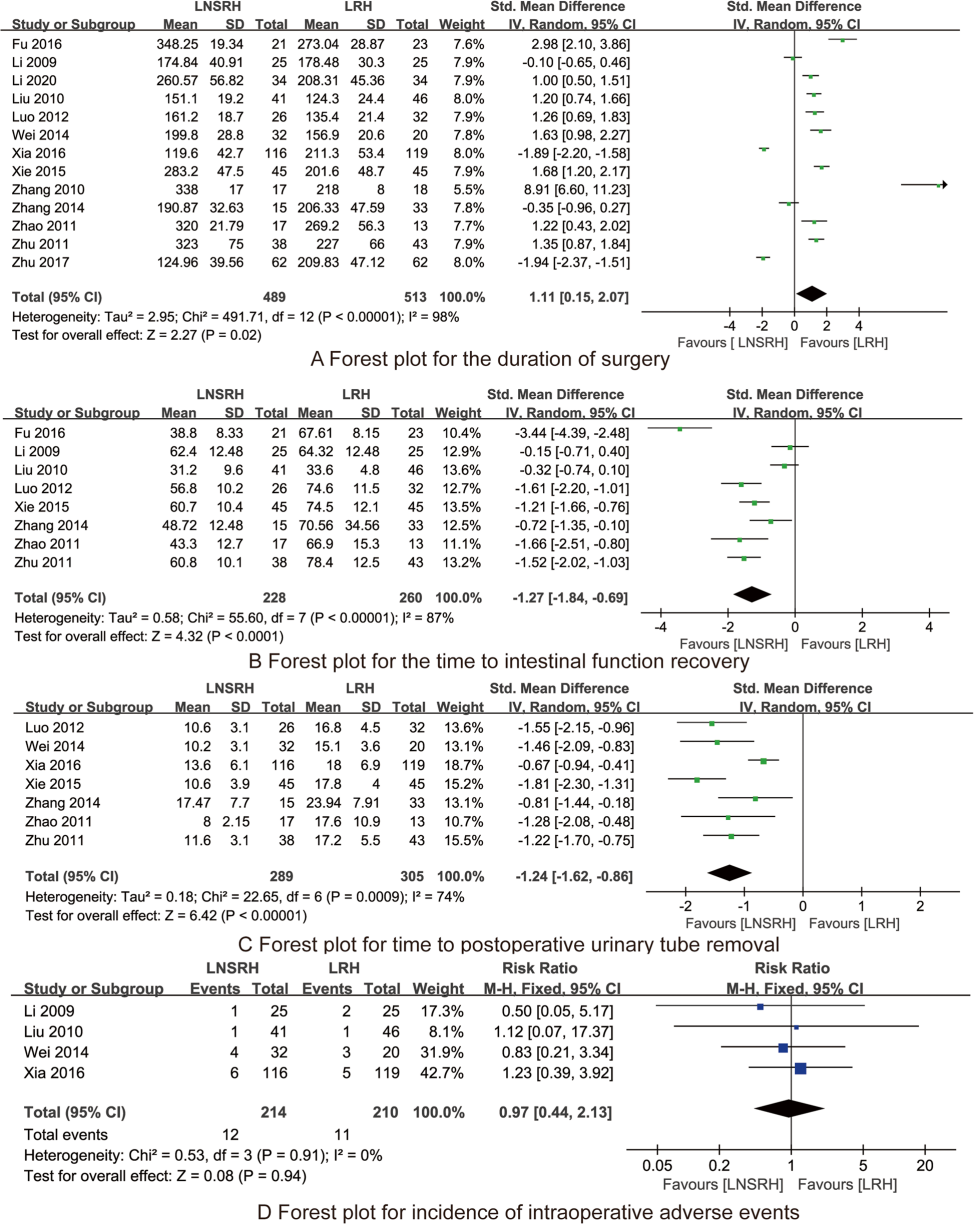
The forest plots for synthesized outcomes

All 13 RCTs [13–25] reported the duration of surgery. As shown in Fig. 5A, the duration of surgery of the LNSRH group is longer than that of the LRH group, and the difference was statistically significant [SMD 1.11, 95% CI (0.15 ~ 2.07), *P* = 0.02].

#### Time to intestinal function recovery

All 8 RCTs [14–19, 22, 24] reported the time to intestinal function recovery. As shown in Fig. 5B, the time to intestinal function recovery of the LNSRH group is less than that of the LRH group, and the difference was statistically significant [SMD −1.27, 95% CI (−1.84 ~ −0.69), *P* < 0.001].

#### Time to postoperative urinary catheter removal

All 7 RCTs [13–17, 20, 24] reported the time to postoperative urinary catheter removal. As shown in Fig. 5C, the time to postoperative urinary catheter removal of the LNSRH group is less than that of the LRH group, and the difference was statistically significant [SMD −1.24, 95% CI (−1.62 ~ −0.86), *P* < 0.001].

#### Incidence of intraoperative adverse events

All 4 RCTs [13, 18–20] reported the incidence of intraoperative adverse events between LNSRH group and the LRH group. As presented in Fig. 5D, there is no significant difference in the incidence of intraoperative adverse events between the LNSRH group and the LRH group [RR 0.97, 95% CI (0.44 ~ 2.13), *P* = 0.94].

### Publication bias

**Fig. 6:**
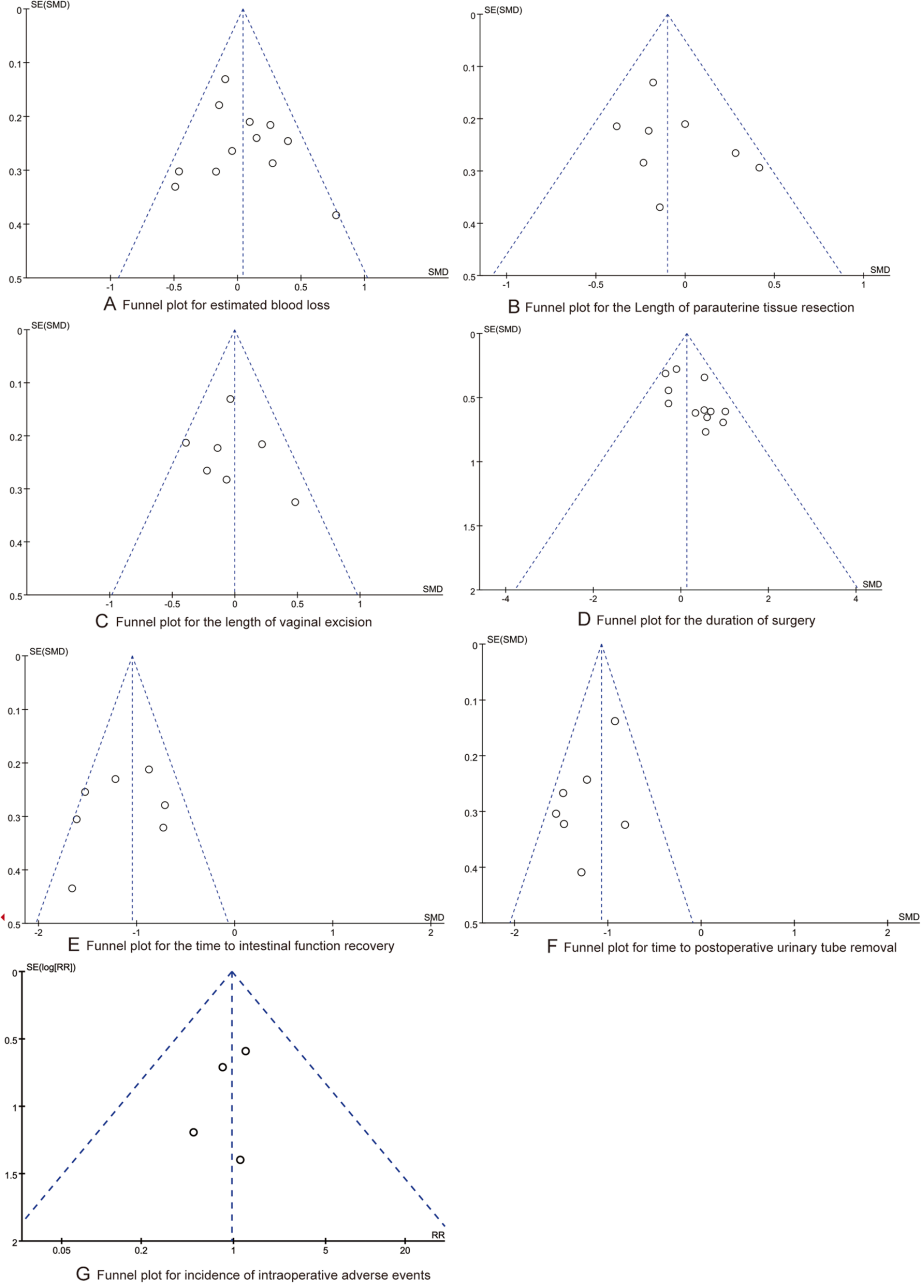
The funnel plots of synthesized outcomes

We evaluated the publication biases by using a funnel plot, as shown in Fig. 6, all the dots are evenly distributed in the funnel plots for every synthesized outcome. And the results of Egger regression test indicated that there were no significant publications (all *P* > 0.05).

Sensitivity analyses, which investigate the influence of single one study on the overall risk estimate by removing RCT one by one, found that the overall risk estimates were not substantially changed by any single RCT.

## Discussions

As a surgical procedure for the clinical treatment of patients with early cervical cancer, LRH has multiple advantages such as less intraoperative blood loss, less trauma, and rapid postoperative recovery compared with traditional open surgery without compromising the extend of surgical resection [26]. It has been discovered that the minimally invasive advantages of laparoscopy of laparoscopic magnification enable identification of the pelvic view and identify the pelvic autonomic nerves and resulting in its preservation [27]. The results of this study have suggested that LNSRH takes a longer time compared with LRH, mainly because this operation requires the surgeon to be meticulous, and the pelvic autonomic nerve needs to be carefully identified and isolated during the operation to protect the pelvic autonomy nerve [26]. The duration of LNSRH is longer than LRH, but with advancement of operative skills, the procedure can be optimized and the operation time can be shortened [28].

In our meta-analysis, we have found that LNSRH is beneficial to improve the postoperative bladder and intestinal function recovery when compared with LRH. Radical cervical cancer surgery requires the removal of the sacral ligament, the cardinal ligament, and the upper 1/3 of the vagina at the level close to the pelvic wall [29, 30]. Some of pelvic autonomic nerves damaged resulting in postoperative reduction in bladder compliance, insufficiency of bladder neck closure, and urinary incontinence. The pelvic splanchnic nerve plexus is dominated by the parasympathetic nerve [27]. Clearing the lymph nodes around the internal iliac vein and the deep uterine vein may result in damage and a decrease in the sensitivity of the bladder to pressure and causing bladder dysfunction [31]. Damage to the rectal branch of the pelvic plexus can cause rectal dysfunction [32]. Usually, these complications would partially improve over time, but there are still a considerable proportion of patients who will not; this seriously affects their quality of life [33]. In recent years, the large-scale promotion of laparoscopic technology and the successful development of new instruments have provided technical support for the implementation of nerve-sparing surgery. In the past 10 years, radical resection of cervical cancer with preservation of pelvic visceral nerves has become a topic of interest in the field of gynecological oncology, and reports on its effectiveness and safety have been increasing. The preservation of pelvic nerves is beneficial to the recovery of bladder and rectal function [34]. Most studies have shown that patients undergoing LNSRH have no significant differences with LRH in terms of the number of lymph nodes to be removed, the length of parauterine tissue resection, and the length of vaginal tissue resection [35–37]. However, LNSRH is better than LRH in terms of postoperative bladder and intestinal function recovery, which is consistent with our findings.

In this study, there is no difference between LNSRH and LRH in terms of intraoperative blood loss, length of parauterine tissue resection, and length of vaginal resection. However, LNSRH is longer than LRH in terms of duration of surgery. It is generally believed that in LNSRH, the pelvic autonomic nerves are preserved without sacrificing the radicality of surgery [38]. There is currently no uniform standard to evaluate that the success of nerve preservation is achieved [39]. Two methods are commonly used for evaluation of bladder function by comparing the preoperative and postoperative urodynamic parameters or the duration of postoperative catheter indwelling time [40]. There is no significant difference in the two procedures with regard to length of parametrial tissue excised and the length of vagina removed, it may be explained that both LNSRH and LRH are minimally invasive surgeries, the incisions are aimed to be as small as possible in clinical practice [41, 42]. And the sample size included may be not enough to detect the potential differences length of parametrial tissue excised and the length of vagina. It is generally believed that a successful LNSRH results in a catheter indwelling time of about 10 days [43]. The results of this study have confirmed the effect of preserving pelvic autonomic nerves on reducing bladder dysfunction. However, it is an alternative laparoscopic method to evaluate successful nerve preservation on the postoperative catheter removal time [44]. Currently, there are very few reports on comparing preoperative and postoperative urodynamic parameters to evaluate the surgical effect and safety. More prospective and well-designed researches on this issue are needed in the future.

This meta-analysis has certain limitations that must be highlighted. Firstly, the included RCTs are all Chinese studies, and there is a lack of RCT research reports from other countries and populations. The results of the research may be subjected to bias by region and population. Secondly, related results such as operation time and intraoperative blood loss are heterogeneous, and this may be related to the variable skill level of individual surgeons. Thirdly, there was only one of the 13 included articles reported postoperative survival, and there was a lack of effective analysis of the long-term effects of surgery in different stage of cervical cancer. Future studies are needed to further explore the long-term effects of LNSRH.

## Conclusions

In conclusion, the results of this meta-analysis show that LNSRH has its unique advantages in the treatment of cervical cancer. It significantly results in earlier recovery of bladder and intestinal function of patients after surgery without compromising the curative effect of surgery, which has application prospects and development trends in clinical cervical cancer treatment. However, the research areas and populations included in this meta-analysis are limited. Multi-center, large-sample RCTs are needed for in-depth evaluation of effectiveness and safety of LNSRH, and to provide reliable evidence to advance surgical cervical cancer treatment.

## Data Availability

All data generated or analyzed during this study are included in this published article.

## Abbreviations

LNSRH: Laparoscopic nerve‑sparing radical hysterectomy
LRH: Laparoscopic radical hysterectomy
RCTs: Randomized controlled trials
PRISMA: Preferred Reporting Items for Systematic Reviews and Meta-Analyses
CNKI: China National Knowledge Infrastructure
RR: Risk ratio
CI: Confidence intervals
SMD: Standardized mean differences
